# Predictive value of pentraxin-3 on disease severity and mortality risk in patients with hemorrhagic fever with renal syndrome

**DOI:** 10.1186/s12879-021-06145-0

**Published:** 2021-05-17

**Authors:** Hong Du, Haifeng Hu, Pingzhong Wang, Xiaoyan Wang, Ying Zhang, Hong Jiang, Jing Li, Xuefan Bai, Jianqi Lian

**Affiliations:** Center for Infectious Diseases, Second Affiliated Hospital of Air Force Medical University, 569 Xinsi Rd, Baqiao District, Xi’an, 710038 Shaanxi China

**Keywords:** Hemorrhagic fever with renal syndrome, Pentraxin-3, Early prediction, Prognosis, Disease severity

## Abstract

**Background:**

Hemorrhagic fever with renal syndrome (HFRS) caused by Hantaan virus is characterized by systemic immunopathological injury. Pentraxin-3 is an acute-phase reactant involved in the processes of inflammation and infection. This study aimed to investigate the levels of plasma pentraxin-3 and evaluate its predictive value on disease severity and mortality risk in patients with HFRS.

**Methods:**

This was a prospective real-world observational study. The concentrations of plasma pentraxin-3 were measured by enzyme linked immunosorbent assay (ELISA) in 105 HFRS patients and 27 healthy controls. We analyzed the clinical relevance between pentraxin-3 and clinical subtyping, hospital stay and conventional laboratory parameters of HFRS patients. Considering the prognosis (death) as the primary endpoint, the levels of pentraxin-3 between survivors and non-survivors were compared, and its association with mortality was assessed by Kaplan-Meier survival analysis. The predictive potency of pentraxin-3 for mortality risk in HFRS patients was evaluated by receiver operating characteristic (ROC) curve analysis.

**Results:**

The levels of pentraxin-3 during the acute phase were increased with the aggravation of the disease, and showed the highest expression in critical-type patients (*P* < 0.05). Pentraxin-3 demonstrated significant correlations with conventional laboratory parameters (WBC, PLT, AST, ALB, APTT, Fib) and the length of hospital stay. Compared with the survivors, non-survivors showed higher levels of pentraxin-3 and worse expressions of conventional laboratory parameters during the acute phase. The Kaplan-Meier survival curves showed that high levels of pentraxin-3 during the acute phase were significantly associated with the death in HFRS patients. Pentraxin-3 demonstrated significant predictive value for the mortality risk of HFRS patients, with the area under ROC curve (AUC) of 0.753 (95%*CI*: 0.593 ~ 0.914, *P* = 0.003).

**Conclusions:**

The detection of plasma pentraxin-3 might be beneficial to the evaluation of disease severity and to the prediction of mortality risk in HFRS patients.

## Background

Hemorrhagic fever with renal syndrome (HFRS) is a kind of natural focus disease caused by Hantavirus infection, and characterized by fever, hemorrhage and renal impairment [1]. Hantavirus infection could induce the destruction of vascular endothelial cells [2], the diffuse damage of systemic micro-vessels, the increase of capillary permeability and the decrease of platelets [3]. Patients with intemperate immunoreaction may further develop “capillary leakage syndrome”, which could result in secondary edema, hypovolemic shock, acute kidney injury (AKI), coagulation disorder and even multiple organ dysfunction syndrome (MODS) [4]. It has been accepted that HFRS has immunopathological features of systemic inflammatory response syndrome (SIRS) [5]. The early diagnosis and disease severity assessment may help the clinicians choose the best therapeutic schedule for patients and finally improve the therapeutic effect of HFRS [6]. Given the limited predictive value of the conventional laboratory parameters, it is necessary to explore some novel biomarkers for evaluating the severity and prognosis in patients with HFRS.

As the first long-pentraxin discovered in human, pentraxin-3 is mainly produced by monocyte macrophages and myeloid dendritic cells stimulated by pro-inflammatory signals such as IL-1β, TNF-α and Toll-like receptor activation. Additionally, a fraction of pentraxin-3 is derived from the secretion of neutrophils, lymphocytes and endothelial cells [7]. Pentraxin-3 plays an important role in innate humoral immune response, inflammatory response, anti-infection, as well as tissue damage and repair [8]. Many previous studies have demonstrated that the levels of plasma pentraxin-3 were positively related to the severity of sepsis, acute pancreatitis, acute myocardial injury and other diseases, which indicated that pentraxin-3 can serve as a novel biomarker for inflammation, infection and tissue damage [9–14]. Additionally, the over-expressed pentraxin-3 of neutrophils might be associated with the overproduction of reactive oxygen species (ROS) and vascular endothelial dysfunction. Excessive pentraxin-3 might represent an emerging biomarker for the progression of vascular injury in patients with hemodialysis [15]. Previous studies have also shown that high levels of plasma pentraxin-3 were associated with the development of dengue shock syndrome caused by dengue virus. Pentraxin-3 seemed to be an early prognostic indicator of disease severity in dengue hemorrhagic fever [16]. Outinen et al. [17] showed that high levels of plasma pentraxin-3 were associated with the thrombocytopenia in Puumala hantavirus (PUUV) induced nephropathia epidemica (a mild form of HFRS, mainly prevalent in the Europe). Recent study also indicated that plasma pentraxin-3 was highly correlated with PLT, Fib, APTT and other coagulation indicators, and which got a favorable predictive value for disease severity in patients with nephropathia epidemica [18]. However, the role of pentraxin-3 in HFRS caused by Hantaan virus (HTNV) has not been reported. Given the above research background, we detected the levels of plasma pentraxin-3 in patients with HFRS, and investigated its predictive value for disease severity and prognosis (death) of HFRS.

## Methods

### Study population

We prospectively recruited 105 HFRS patients who were treated in the Second Affiliated Hospital of Air Force Medical University from October 2012 to December 2014. All the enrolled patients met the following inclusion criteria: (1) Consistent with the typical clinical manifestations of HFRS and confirmed by serological examination; (2) 18–70 years old; (3) In the acute phase at the time of hospital admission. In addition, 27 healthy volunteers were recruited as controls. The patients according with any of the following criteria were excluded: (1) Age > 70 or < 18 years; (2) Entered the diuretic stage on admission; (3) Complicated with pregnancy, chronic kidney diseases, autoimmune diseases, bacterial infection, viral hepatitis or other infectious diseases. The study was approved by the Ethics Committee of the Second Affiliated Hospital of Air Force Medical University and the written informed consents were obtained from the participants or their guardians.

### Procedures and definitions

The serological diagnosis of HFRS was confirmed by the positive results of HTNV-specific IgM/IgG capture ELISA. According to the clinical classification criteria of HFRS [19], all the enrolled patients were divided into the following four groups: mild-type, moderate-type, severe-type and critical-type. The specific clinical typing criteria were showed in Table 1. The classification of all patients got a confirmation from their respective attending physician. Furthermore, based upon the typical clinical characteristics of HFRS, we also divided the clinical course into the acute phase (including the febrile, hypotensive, and oliguric stages) and the convalescent phase (including the diuretic and convalescent stages) [20]. The prognosis was defined as survival or death during hospitalization.

**Table 1:** Grouping criteria based upon the clinical typing criteria of HFRS^a^

Group	Clinical typing criteria of HFRS
Mild-type	Patients with slight symptoms and mild renal impairment (proteinuria or hematuria ranging from “+” to “++”), and without obvious oliguria and hypotension.
Moderate-type	Patients with typical symptoms of effusion (bulbar conjunctiva) and petechiae (skin and mucous membranes), and also meeting with obvious oliguria and kidney injury (proteinuria or hematuria more than “+++”), some of them presenting with transient hypotension.
Severe-type	Patients presenting with the following symptoms: (1) severe effusion (bulbar conjunctiva and either pleura orperitoneum) and haemorrhage (skin and mucous membranes); (2) significant hypotension and uremia;(3) AKI with oliguria (urine output 100 ~ 500 mL/day) ≤5 days or anuria (urine output < 100 mL/day) ≤2 days.
Critical-type	On the basis of the severe-type, patients meeting with any of the following complications: refractory shock (≥2 days), visceral hemorrhage, heart failure, pulmonary edema, brain edema, severe secondary infection, and severe AKI with either oliguria (urine output 100 ~ 500 mL/day) > 5 days or anuria (urine output < 100 mL/day) > 2 days.

*Abbreviation*: *AKI* Acute kidney injury

^a^The clinical typing criteria of HFRS was derived from the “Prevention and Treatment Strategy of Hemorrhagic Fever with Renal Syndrome” promulgated by the National Health Commission of China

### Pentraxin-3 detection and clinical data collection

Venous blood samples of the patients were collected once each in the acute phase and convalescent phase, and were centrifuged (1200 g, 10 min, 4 °C) within 2 h after drawing. Pentraxin-3 levels of supernatant plasma were measured with commercially available ELISA kits (Quantikine, XiTang Inc., Shanghai, China). Conventional laboratory tests (blood routine test, biochemical test and blood condensation test) were performed in the clinical laboratory as ordered by attending physicians. Data of demographic, duration of hospital stay, clinical outcome (discharge or death) and conventional laboratory parameters (blood sampling time was the same as that of pentraxin-3) were extracted from the electronic medical records.

### Statistical analysis

The study was initially designed to compare the difference of plasma pentraxin-3 levels between HFRS patients and healthy controls. Our primary hypothesis was that the levels of plasma pentraxin-3 in HFRS patients would significantly higher than in healthy controls. Based upon the results of our pre-experiment, the assumed mean concentration of plasma pentraxin-3 in HFRS patients and healthy controls were 287.3 ng/mL and 8.5 ng/mL, with the standard deviation of 438.5 and 13.6, respectively. Setting a 5:1 ratio of HFRS patients and healthy controls, we calculated that the original target sample size of 132 cases (110 HFRS patients and 22 healthy controls) would provide 81.15% power under a two-sided type I error of 5%.

All the statistical analyses were performed using SPSS 23.0 software (IBM Inc., Chicago IL, USA). Continuous variables of normal distribution were presented as mean ± standard deviation, and were compared by Student’s t-test or one-way analysis of variance. Continuous variables of non-normal distribution were presented as median (interquartile range), and were compared by Mann-Whitney U test or Kruskal-Wallis H test. The categorical variables were presented as numbers (percentage) and were compared by chi-square test. The levels of pentraxin-3 during the acute phase and convalescent phase were compared by Wilcoxon matched-pairs signed-ranks test. Spearman correlation analysis was used to evaluate the correlations of pentraxin-3 with conventional laboratory parameters and hospital stay. The predictive efficacy of pentraxin-3 for the prognosis (death) of HFRS patients was evaluated by the receiver operating characteristic (ROC) curve analysis and quantified by the area under ROC curve (AUC). The comparison of AUC between pentraxin-3 and conventional laboratory parameters was performed using Mann-Whitney U test. Kaplan-Meier survival curves with log-rank test were used to assess the association of laboratory markers with mortality and calculate the hazard ratio (HR) of death. A two-sided *P* < 0.05 was considered statistically significant.

## Results

### Demographic, clinical and laboratory parameters of the study population

From October 2012 to December 2014, a total of 386 HFRS patients were treated in our center. Of them, excluding the patients who did not meet the inclusion criteria (92 cases) and the patients who refused to participate in the study (189 cases), 105 patients with a mean age of 41.85 ± 15.35 years were enrolled in this prospective observational study, including 21 (20.0%) females and 84 (80.0%) males.

According to the grouping criteria mentioned above, 17 cases were classified as mild-type, 27 cases were classified as moderate-type, 26 cases were classified as severe-type, and 35 cases were classified as critical-type. Of all the enrolled patients, 14 (13.3%) critical-type patients were died and the rest were recovered and discharged. There was no significant difference in gender, age distribution, and the timing of blood collection during the acute phase among the subgroups (*P* > 0.05) (Table 2). The length of hospital stay increased with the aggravation of the disease, while not longest in the critical-type patients, which attributed to the fact that the death usually occurred soon after admission in the non-survivors. Excluding the non-survivors, the length of hospital stay showed a significant correlation with disease severity (Table 2).

**Table 2:** Demographic, clinical and laboratory parameters of the study population

	Grouping according to the clinical type							Grouping according to the prognosis			
	Mild-type (***n*** = 17)	Moderate-type (***n*** = 27)	Severe-type (***n*** = 26)	Critical-type (***n*** = 35)	Healthy controls (***n*** = 27)	***χ***^***2***^***/F/H***	***P*** value	Survivors (***n*** = 91)	Non-survivors (***n*** = 14)	***χ***^***2***^***/t/Z***	***P*** value
Gender											
Female	6 (35.3%)	5 (18.5%)	3 (11.5%)	7 (20.0%)	6 (22.2%)	3.690	0.450	17 (20.7%)	3 (21.4%)	0.004	0.953
Male	11 (64.7%)	22 (81.5%)	23 (88.5%)	28 (80.0%)	21 (77.8%)			65 (79.3%)	11 (78.6%)		
Age, years	36.47 ± 17.14	40.07 ± 13.67	43.62 ± 12.96	44.51 ± 15.09	40.65 ± 13.16	1.419	0.242	41.76 ± 14.34	46.57 ± 16.52	−1.136	0.259
^a^ Timing, days	7.0 (5.0–7.5)	6.0 (5.0–7.0)	6.0 (5.0–7.0)	6.0 (5.0–8.0)	–	1.036	0.793	7.0 (5.0–8.0)	6.0 (4.5–6.5)	−1.643	0.058
Hospital stay, days											
All patients	10.00 ± 2.15	13.72 ± 3.41	20.42 ± 5.95	17.32 ± 11.81	–	6.771	< 0.001	16.0 (12.0–23.0)	6.0 (1.76–10.5)	−4.999	< 0.001
Survivors	10.00 ± 2.15	13.72 ± 3.41	20.42 ± 5.95	26.41 ± 7.13	–	34.549	< 0.001				
Pentraxin-3, ng/mL											
Acute phase	58.21 (22.84–334.37)	216.13 (63.61–539.01)	171.07 (51.28–458.82)	570.30 (138.37–1088.51)	0.46 (0.18–0.65)	69.128	< 0.001	181.79 (41.14–518.53)	935.04 (258.74–1453.42)	−3.021	0.003
Convalescent phase	2.52 (1.25–11.19)	11.30 (5.64–26.90)	5.87 (3.14–8.00)	4.92 (2.49–11.34)		56.540	< 0.001	–	–	–	–
WBC, × 10^9^/L	11.60 (7.86–13.34)	11.08 (7.67–16.72)	13.37 (8.91–20.16)	14.95 (9.62–27.72)	–	6.019	0.111	12.49 (8.45–18.15)	33.05 (12.04–46.58)	−2.886	0.004
PLT, ×10^9^/L	58.50 (50.25–104.50)	37.00 (25.50–55.50)	34.00 (22.75–51.50)	26.00 (14.00–54.50)	–	11.966	0.008	39.50 (24.00–58.25)	22.50 (10.25–39.50)	−2.938	0.003
ALB, g/L	31.31 ± 3.96	29.30 ± 4.43	28.22 ± 6.01	27.17 ± 4.67	–	2.008	0.120	28.86 ± 5.06	23.81 ± 8.21	2.227	0.042
ALT, U/L	37.50 (28.75–59.00)	43.00 (28.25–68.75)	53.00 (42.75–88.50)	64.00 (49.00–118.00)	–	8.149	0.043	51.00 (32.00–78.50)	97.00 (55.50–265.50)	−3.081	0.002
AST, U/L	69.50 (47.25–105.50)	81.00 (56.00–112.75)	89.50 (60.50–154.00)	121.00 (69.00–197.00)	–	5.582	0.134	85.00 (59.00–124.00)	260.50 (185.00–825.75)	−4.543	< 0.001
PT, sec	10.90 (10.63–11.70)	11.50 (9.75–12.20)	11.50 (10.60–13.05)	11.50 (10.80–13.20)	–	1.710	0.635	11.50 (10.60–12.40)	15.10 (12.53–21.00)	−3.982	< 0.001
APTT, sec	34.22 ± 5.02	37.17 ± 7.48	41.22 ± 9.77	35.49 ± 8.03	–	2.873	0.042	36.00 (31.90–43.00)	58.50 (41.95–75.20)	−4.341	< 0.001
Fib, g/L	2.97 ± 0.91	2.49 ± 0.72	2.77 ± 1.02	2.26 ± 1.01	–	1.861	0.144	2.62 ± 0.93	1.54 ± 0.76	4.104	< 0.001
BUN, mmol/L	8.86 ± 4.92	14.54 ± 9.06	19.55 ± 11.01	19.78 ± 6.94	–	5.699	0.001	13.97 (7.07–22.99)	13.27 (7.61–29.90)	−0.426	0.670
Cr, μmol/L	112.40 (66.15–213.65)	175.60 (85.50–333.90)	303.10 (122.40–540.15)	431.30 (248.65–533.00)	–	16.075	0.001	216.35 (109.63–481.75)	245.25 (97.10–327.78)	−0.405	0.686

*Abbreviations*: *WBC* White blood cells, *PLT* Platelets, *ALB* Albumin, *ALT* Alanine aminotransferase, *AST* Aspartate aminotransferase, *PT* Prothrombin time, *APTT* Activated partial thromboplastin time, *Fib* Fibrinogen, *BUN* Blood urea nitrogen, *Cr* Creatinine, *sec* Second

^a^Timing: Timing of blood collection druingthe acute phase, duration from illness onset to the day of blood collection

**Fig. 1:**
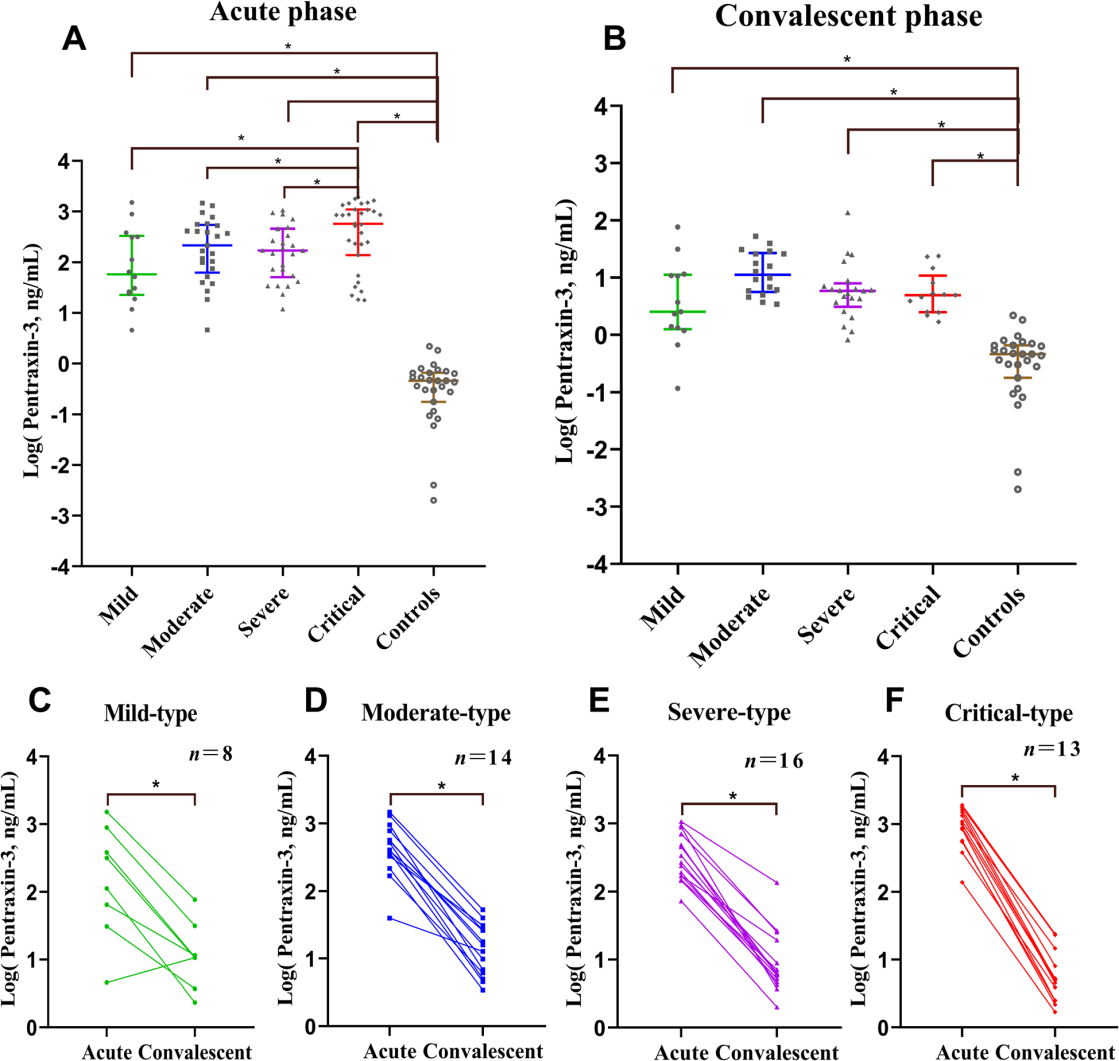
Levels of plasma pentraxin-3 during the clinical course in patients with HFRS The levels of plasma pentraxin-3 during the acute phase (**a**) and the convalescent phase (**b**) were compared by Kruskal-Wallis H test; pairwise comparisons among the five groups were performed using the Nemenyi rank test. The differences of plasma pentraxin-3 between acute phase and convalescent phase of the mild-type (**c**), the moderate-type (**d**), the severe-type (**e**), and the critical-type (**f**) were compared by Wilcoxon matched-pairs signed-ranks test. The logarithmic value of pentraxin-3 was used in the figure for the reason of the great differences between the groups.* *P* < 0.05

We comparatively analyzed the levels of pentraxin-3 among the subgroups to investigate the clinical correlation of pentraxin-3 with disease severity. Of the enrolled patients, the levels of pentraxin-3 during the acute phase were significantly higher than that of the control group and convalescent phase of the same type (*P* < 0.05). The levels of pentraxin-3 had an increasing tendency with the aggravation of the disease, and showed the highest expression in critical-type patients (*P* < 0.05). By contrast, the comparison of pentraxin-3 during the convalescent phase among the four types demonstrated no significant difference (*P* < 0.05) (Fig. 1). Compared with the survivors, non-survivors got higher levels of pentraxin-3 and worse expressions of the conventional laboratory parameters (except for the renal function markers: blood urea nitrogen and creatinine) during the acute phase. More detailed information about demographic, clinical and laboratory parameters of the study population was displayed in Table 2.

### The correlations of pentraxin-3 with conventional laboratory parameters and hospital stay

**Fig. 2:**
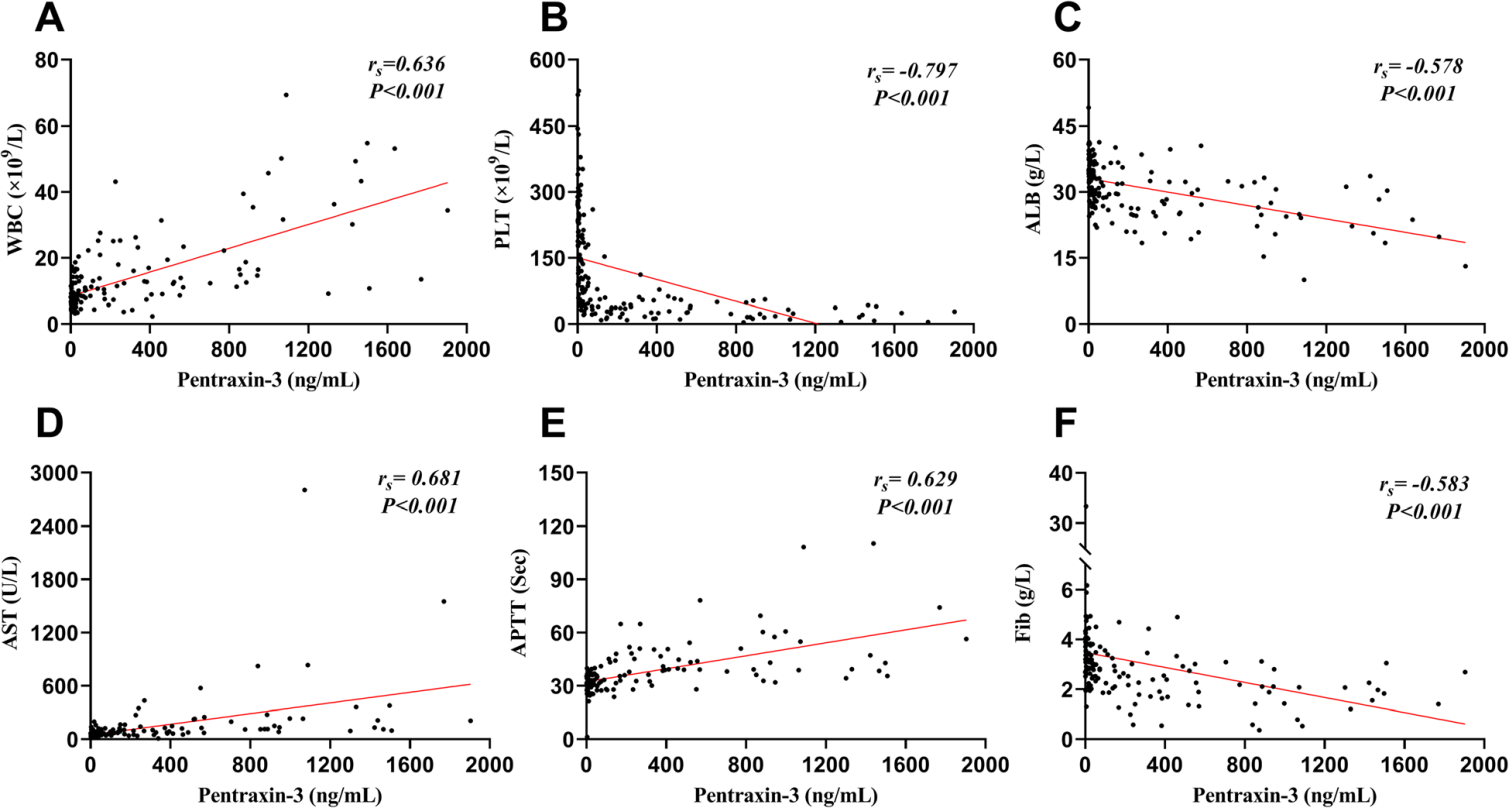
The correlation between pentraxin-3 and conventional laboratory parameters. Figure shows the correlation between pentraxin-3 and WBC (**a**), PLT (**b**), ALB (**c**), AST (**d**), APTT (**e**), Fib (**f**). Spearman rank correlation analysis was used to evaluate the correlation between pentraxin-3 and conventional laboratory parameters. Abbreviations: WBC, White blood cells; PLT, Platelet; AST, Aspartate aminotransferase; ALB, Albumin; APTT, Activated partial thromboplastin time; Fib, Fibrinogen

Spearman correlation analysis showed that pentraxin-3 was positively correlated with WBC, AST and APTT, and negatively correlated with PLT, ALB and Fib (**|***r*_*s*_**|** > 0.500, *P* < 0.001) (Fig. 2). With hospital stay as a clinically relevant end point, pentraxin-3 and conventional laboratory parameters (WBC, PLT, AST, BUN, Cr) showed significant correlations with the length of hospital stay (Table 3).

**Table 3:** ^a^Correlations of the laboratory parameters with pentraxin-3 and hospital stay

Laboratory parameters	Pentraxin-3, ng/mL		^**b**^ Hospital stay, days	
	***r***_***s***_	***P*** value	***r***_***s***_	***P*** value
Pentraxin-3, ng/mL	–	–	0.232	0.039
WBC, × 10^9^/L	0.636	< 0.001	0.259	0.019
PLT, ×10^9^/L	−0.797	< 0.001	− 0.323	0.003
HGB, g/L	0.385	< 0.001	0.057	0.614
HCT, %	0.265	0.001	−0.031	0.784
ALB, g/L	−0.578	< 0.001	−0.180	0.107
ALT, U/L	0.303	< 0.001	0.172	0.135
AST, U/L	0.681	< 0.001	0.249	0.029
PT, sec	0.155	0.065	0.208	0.066
PTA, %	−0.134	0.112	−0.218	0.053
APTT, sec	0.629	< 0.001	0.201	0.076
Fib, g/L	−0.583	< 0.001	− 0.156	0.169
BUN, mmol/L	0.203	0.010	0.459	< 0.001
Cr, μmol/L	−0.040	0.618	0.454	< 0.001
UA, μmol/L	−0.214	0.006	0.016	0.889

*Abbreviations*: *r*_*s*_ Coefficient of rank correlation, *WBC* White blood cells, *PLT* Platelets, *HGB* Hemoglobin, *HCT* Hematocrit, *ALB* Albumin, *ALT* Alanine aminotransferase, *AST* Aspartate aminotransferase, *PT* Prothrombin time, *PTA* Prothrombin activity, *APTT* Activated partial thromboplastin time, *Fib* Fibrinogen, *BUN* Blood urea nitrogen, *Cr* Creatinine, *UA* Uric acid, *sec* Second

^a^The correlations were calculated by Spearman rank correlation analysis

^b^The data of hospital stay was derived from the survivors

### ROC curves for predictive efficacy and the hazard ratio of death

**Fig. 3:**
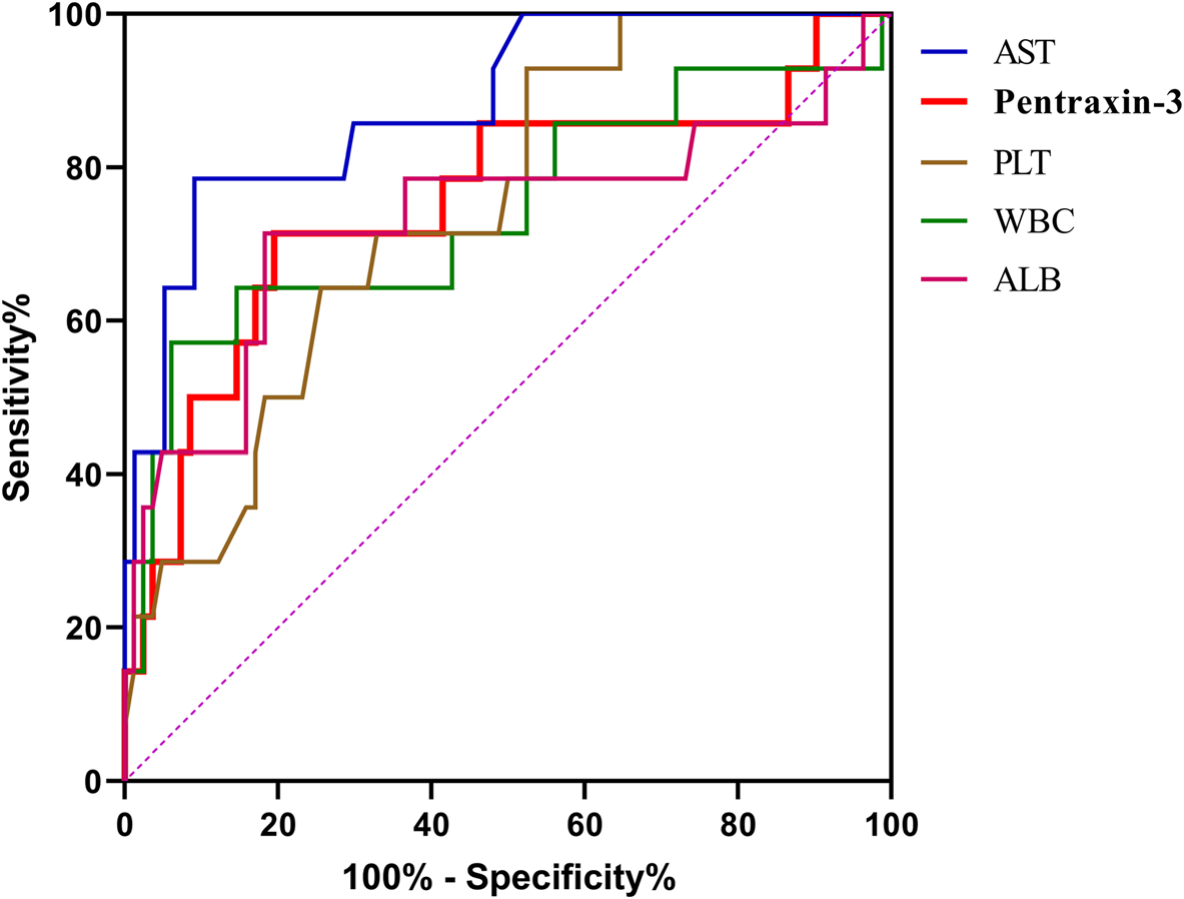
ROC curves for evaluating the predictive efficacy of pentraxin-3 and conventional laboratory parameters. Figure shows the predictive efficacy of pentraxin-3, AST, PLT, WBC, and ALB for the prognosis (death) in patients with HFRS. Pentraxin-3 showed significant predictive value, with the AUC of 0.753 (*P* = 0.003). Abbreviations: ROC curve, Receiver operating characteristic curve; AUC, Area under the ROC curve; WBC, White blood cells; PLT, Platelet; AST, Aspartate aminotransferase; ALB, Albumin

The results of ROC curve analysis demonstrated obvious predictive value of pentraxin-3 for the prognosis (death) of HFRS patients, with the AUC of 0.753 (95%*CI*: 0.593 ~ 0.914, *P* = 0.003) (Fig. 3). Except for AST, the AUC of pentraxin-3 demonstrated no significant difference with that of conventional laboratory parameters (WBC, PLT, ALB, APTT and Fib). Taking 569.088 ng/ml as the cut-off value of pentraxin-3, the sensitivity and specificity of pentraxin-3 for predicting the prognosis (death) was 71.4 and 80.5%, respectively (Table 4).

**Table 4:** Predictive efficacy of pentraxin-3 and conventional laboratory parameters

	AUC (95% ***CI***)	***P*** value	Cut-off value	Sensitivity	Specifity	Comparison of AUC (pentraxin-3)	
						***Z***	***P*** value
Pentraxin-3, ng/mL	0.753 (0.593–0.914)	0.003	569.088	71.4%	80.5%	–	–
WBC, ×10^9^/L	0.742 (0.573–0.911)	0.004	31.525	57.1%	93.9%	0.188	0.851
PLT, × 10^9^/L	0.747 (0.621–0.872)	0.003	41.5	47.6%	92.9%	0.110	0.912
ALB, g/L	0.732 (0.553–0.911)	0.006	24.85	81.7%	71.4%	0.344	0.731
AST, U/L	0.883 (0.787–0.979)	< 0.001	203	78.6%	90.9%	2.163	0.031
APTT, sec	0.865 (0.747–0.984)	< 0.001	54.55	64.3%	96.2%	1.670	0.095
Fib, g/L	0.824 (0.710–0.937)	< 0.001	1.8425	86.1%	71.4%	0.785	0.433
Cr, μmol/L	0.534 (0.391–0.677)	0.686	–	–	–	–	–
BUN, mmol/L	0.536 (0.359–0.712)	0.670	–	–	–	–	–

*Abbreviations*: *AUC* Area under the ROC curve, *CI* Confidence interval, *WBC* White blood cells, *PLT* Platelets, *ALB* Albumin, *AST* Aspartate aminotransferase, *APTT* Activated partial thromboplastin time, *Fib* Fibrinogen, *Cr* Creatinine, *BUN* Blood urea nitrogen, *sec* Second

**Fig. 4:**
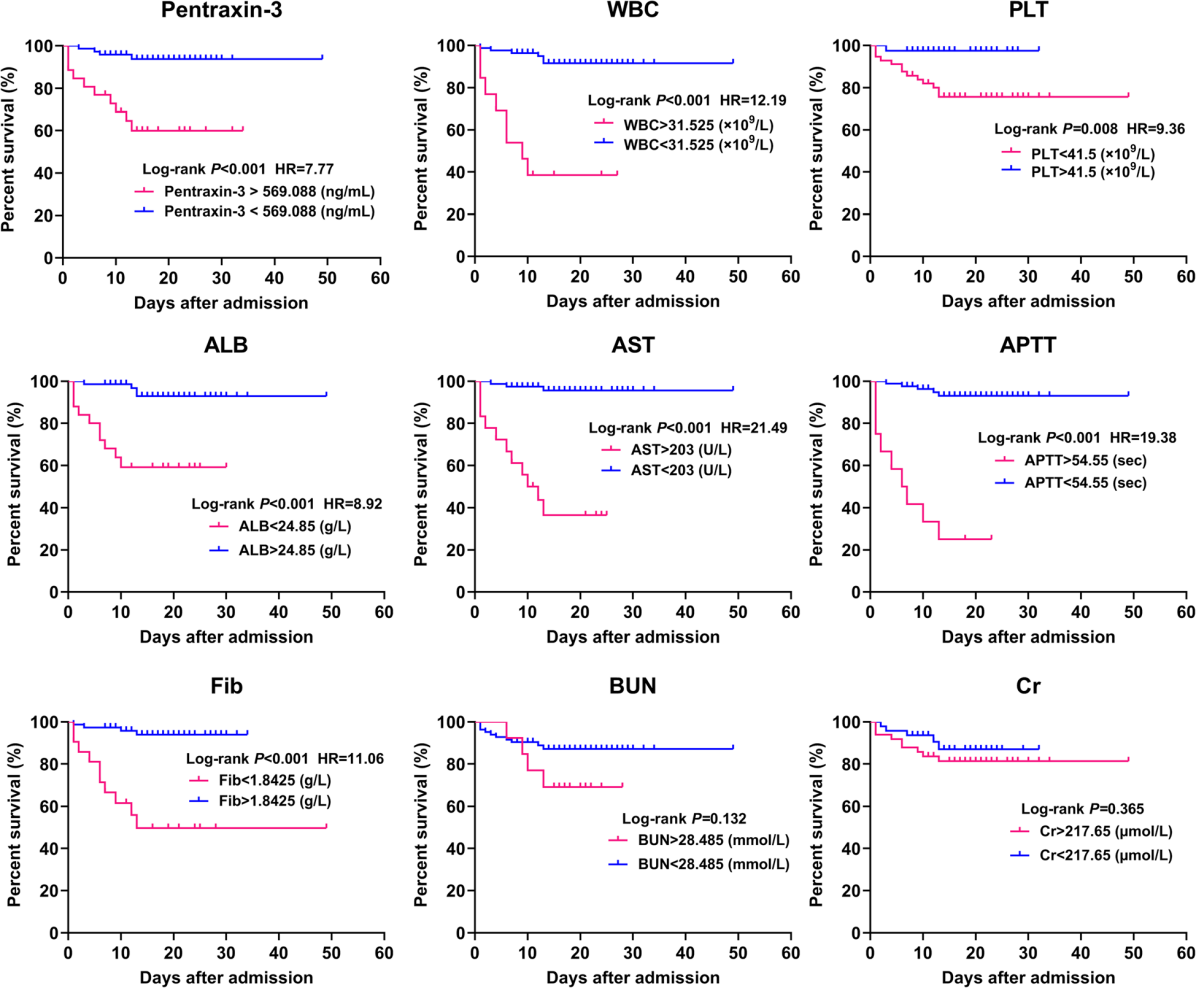
Kaplan-Meier survival curves of pentraxin-3 and conventional laboratory parameters. Figure shows the association of laboratory parameters (pentraxin-3, WBC, PLT, ALB, AST, APTT, Fib, BUN and Cr) with mortality, as well as the death hazard ratio (HR) of each parameter

Furthermore, we assessed the association of laboratory parameters with mortality and calculated the hazard ratio (HR) of death using the Kaplan-Meier survival curves and log-rank test (taking death/survival as the terminal event, lengths of hospital stay as the survival time, and grouping based upon the cut-off values of ROC curves). The results showed that high levels of pentraxin-3 (> 569.088 ng/mL) during the acute phase were significantly associated with the death in HFRS patients, with a HR of 7.77 (2.29–26.35) in comparison with the low pentraxin-3 (< 569.088 ng/mL) (Fig. 4).

## Discussion

Although pentraxin-3 has been well studied in other diseases such as sepsis, acute pancreatitis and dengue hemorrhagic fever [9, 10, 12, 14, 16], its expression and role has not been reported in HTNV-induced HFRS. In this study, we detected the levels of plasma pentraxin-3 in HFRS patients and healthy controls. Our results demonstrated that the expression of plasma pentraxin-3 in HFRS patients was significantly higher than that in healthy controls. Pentraxin-3 levels had an increasing tendency with the aggravation of the disease, and showed the highest expression in the critical-type patients (Table 2 and Fig. 1). The above findings indicate that pentraxin-3 can serve as an early predictor for the disease severity of HFRS, which chimes in with the results of pentraxin-3 in sepsis, acute pancreatitis, nephropathia epidemica and other diseases [9, 10, 12, 14, 16, 17].

Previous studies have indicated that the levels of pentraxin-3 showed significant correlations with coagulation and inflammation parameters in patients with sepsis, nephropathia epidemica, and other infectious diseases [14, 18]. Coincidentally, our study showed that pentraxin-3 was significant correlated with conventional laboratory parameters (WBC, PLT, AST, ALB, APTT, Fib) and the length of hospital stay in HFRS patients (Table 3 and Fig. 2). All the above findings further underline the role of pentraxin-3 on severity assessing in many diseases like HFRS. In addition, our study delivers an explicit result that non-survivors had higher levels of pentraxin-3 and worse expressions of the conventional laboratory parameters during the acute phase (Table 3). However, this was not the case for the markers of kidney function (blood urea nitrogen and creatinine) for the reason that most non-survivors died of refractory shock, rock-ribbed visceral edema and hemorrhage in the febrile and hypotensive stage. It is widely acceptable that the deterioration of kidney function markers is mainly manifested in the relatively stable oliguric stage. Therefore, the markers of kidney function exhibit hysteresis on reflecting the severity of HTNV-induced HFRS [21].

Admittedly, there is a considerable diversity and heterogeneity in the clinical course and prognosis of patients with HFRS. A marker reliably predicting the severity and prognosis of HFRS would be of clinical relevance, since it could help clinicians in individualized therapy allocation based on the disease severity. In recent years, some studies have identified several early predictors related to the severity and prognosis of HFRS, such as growth arrest-specific 6 protein, urinary neutrophil gelatinase-associated lipocalin and Glycoprotein YKL-40 [20–22]. In this study, we observed that high levels of pentraxin-3 (> 569.088 ng/mL) during the acute phase were significantly associated with the death in HFRS patients (Fig. 4). Pentraxin-3 also demonstrated a significant predictive value for the prognosis (death) of HFRS patients, and which was comparable with the predictive value of conventional laboratory parameters such as PLT (Table 4 and Fig. 3). To sum up, pentraxin-3 could serve as a novel and efficient biomarker for predicting the disease severity and mortality risk in patients with HFRS.

As one of the most important immune cells participating in innate immunity, neutrophils play a crucial part in resisting bacterial infection. Nevertheless, the neutrophils are also elevated in most HFRS patients during the acute phase, and also positively correlated with the disease severity of HFRS [19, 23]. Therefore, hyperactive neutrophils may also participate in the immunopathological injury of HFRS by releasing neutrophilic granules. Pentraxin-3 synthesized by neutrophils is mainly stored in neutrophilic granules, which can interact with a variety of bacteria, fungi and viruses after release and then propel the phagocytosis and clearance of pathogenic microorganisms [24]. As an important component of innate humoral immunity, pentraxin-3 has the ability to bind complement component C1q and then activate the classical pathway of complement [25, 26]. Recently, many studies have showed that pentraxin-3 could modulate inflammatory cells, interact with P-selectin, reduce the nitric oxide (NO) synthesis of endothelial cells, inhibit endothelial cells proliferation and alter their functions, and finally promote vascular inflammatory response and endothelial dysfunction [27, 28]. Therefore, the release of pentraxin-3 by neutrophil degranulation may be an important link in the immunopathological injury of HFRS, and the level of plasma pentraxin-3 may also indirectly reflect the severity of vascular endothelial injury in patients with HFRS. However, the underlying pathomechanism of endothelial injury and vascular leakage in HTNV-induced HFRS is still not fully understood. Consequently, further studies are essential to clarify the role of pentraxin-3 on the pathological injury of HFRS and its related mechanism.

As an observational study, although we got a meaningful conclusion that pentraxin-3 could serve as an efficient biomarker for predicting the disease severity and mortality risk of HFRS, there were still some limitations. First, this study was conducted in a single center for infectious diseases. The results might be limited by the relatively small sample size because of the gradually declining incidence of HFRS in Xi’an city. For the reason that several mild-type patients tided over the acute phase instantly after admission and only convalescent samples available, and the decedent died before entering the convalescence phase, only 96 venous blood specimens during the acute phase and 65 during the convalescent phase were collected from the enrolled patients. All these adverse factors may affect the accuracy of the results. Second, the definition of blood sample collection time was too broad in this study. Given the individual differences of patients’ condition and the clinical process on admission, we could only collect venous blood specimens according to the acute phase and convalescent phase defined in the study. Although there was no significant statistical difference on sample collection time of the acute phase, the levels of plasma pentraxin-3 might still be influenced by the different time-points, and also by the variability of pathological injury during the acute phase of HFRS. Third, it is essential to conduct a prospective, large sample, multicenter cohort study to further confirm the predictive efficacy and clinical application value of plasma pentraxin-3 for disease severity and prognosis (death) in HFRS patients. Last but not least, the research limitations caused by the experimental measurement errors and the outdated clinical typing criteria should not be overlooked.

## Conclusions

Pentraxin-3 could serve as a novel and efficient biomarker for predicting the disease severity and mortality risk of patients with HFRS. The detection of plasma pentraxin-3 might help clinicians quickly identify the severe patients at an early stage and timely take optimal therapeutic schedule for them, so as to improve the therapeutic effect and the prognosis of HFRS.

## Data Availability

The datasets used and/or analyzed during the current study are available from the corresponding author on reasonable request.

## Abbreviations

HFRS: Hemorrhagic fever with renal syndrome
AKI: Acute kidney injury
MODS: Multiple organ dysfunction syndrome
SIRS: Systemic inflammatory response syndrome
ROS: Reactive oxygen species
PUUV: Puumala hantavirus
HTNV: Hantaan virus
ELISA: Enzyme linked immunosorbent assay
ROC curve: Receiver operating characteristic curve
AUC: Area under the ROC curve
WBC: White blood cells
PLT: Platelet
HGB: Hemoglobin
HCT: Hematocrit
ALB: Albumin
ALT: Alanine aminotransferase
AST: Aspartate aminotransferase
PT: Prothrombin time
PTA: Prothrombin activity
APTT: Activated partial thromboplastin time
Fib: Fibrinogen
BUN: Blood urea nitrogen
Cr: Creatinine
UA: Uric acid
